# The Role of Thromboinflammation in Delayed Cerebral Ischemia after Subarachnoid Hemorrhage

**DOI:** 10.3389/fneur.2017.00555

**Published:** 2017-10-23

**Authors:** Devin W. McBride, Spiros L. Blackburn, Kumar T Peeyush, Kanako Matsumura, John H. Zhang

**Affiliations:** ^1^The Vivian L. Smith Department of Neurosurgery, McGovern Medical School, The University of Texas Health Science Center at Houston, Houston, TX, United States; ^2^Department of Physiology and Pharmacology, Loma Linda School of Medicine, Loma Linda University, Loma Linda, CA, United States; ^3^Department of Neurosurgery, Loma Linda School of Medicine, Loma Linda University, Loma Linda, CA, United States

**Keywords:** thromboinflammation, delayed cerebral ischemia, subarachnoid hemorrhage, thrombosis, inflammation, cerebral vasospasm

## Abstract

Delayed cerebral ischemia (DCI) is a major determinant of patient outcome following aneurysmal subarachnoid hemorrhage. Although the exact mechanisms leading to DCI are not fully known, inflammation, cerebral vasospasm, and microthrombi may all function together to mediate the onset of DCI. Indeed, inflammation is tightly linked with activation of coagulation and microthrombi formation. Thromboinflammation is the intersection at which inflammation and thrombosis regulate one another in a feedforward manner, potentiating the formation of thrombi and pro-inflammatory signaling. In this review, we will explore the role(s) of inflammation and microthrombi in subarachnoid hemorrhage (SAH) pathophysiology and DCI, and discuss the potential of targeting thromboinflammation to prevent DCI after SAH.

## Introduction

Aneurysmal subarachnoid hemorrhage (aSAH) affects 9 per 100,000 individuals per year in the United States (1) and has high rates of morbidity and mortality (2). Since the mean affected age is 45–55 years old (3), the socioeconomic burden is great, and despite advances in aneurysmal securement technique and management of aSAH in the last 20 years, many of those who survive the initial insult develop significant neurological and cognitive disabilities (1, 4–6).

A major contributor to poor outcomes after aSAH is delayed cerebral ischemia (DCI) (1, 5, 6). DCI affects 20–30% of survivors and occurs between 4 and 10 days after subarachnoid hemorrhage (SAH), leading to cognitive decline (7–9), ultimately causing severe disability and worse quality of life or death (10–12). DCI is the clinical syndrome used to describe delayed development of neurological impairment (9). Historically, DCI was thought to be caused by cerebral vasospasm. However, multiple studies indicate that DCI is multifactorial, with vasospasm being just a contributing factor (13–17) along with cortical spreading depression, disrupted/altered cerebral autoregulation, microthrombosis, and inflammation (6, 18). Due to the multi-faceted nature of DCI involving both inflammation and microthrombosis, in this review, we will provide evidence that thromboinflammation is an unexplored therapeutic target for preventing DCI in aSAH patients.

## SAH Pathophysiology

Following the initial bleed and subsequent treatment of the ruptured aneurysm, the accumulated blood within the subarachnoid space triggers several response mechanisms, as well as pathological events. In response to extravascular blood, endogenous mechanisms of repair are initiated, including removal of the red blood cells (RBCs) (19). To this end, specific cell adhesion molecules are rapidly expressed on the luminal endothelial cell surface (19), which allows circulating macrophages and neutrophils to enter into the subarachnoid space. Extravasated blood is phagocytosed by infiltrating macrophages (20), and as a result, inflammatory cytokines are released by the infiltrated leukocytes and activated resident microglia, stimulating the immune cascade. Indeed, inflammation has been shown to be a major player in early brain injury (21, 22), and may be a factor leading to DCI (23, 24), contributing to long-term deficits (25–27).

A second pathological factor triggered after SAH is hypercoagulability (28). Although the coagulation cascade is immediately activated following aneurysm rupture to stop bleeding, the hypercoagulable state does not end when the bleeding ceases; a sub-set of aSAH patients will continue to have hypercoagulability for several days post-SAH which has been shown to correlate with the development of DCI (28) and result in poorer outcome (28, 29).

### Mechanism of Thromboinflammation after SAH

While there are distinct mechanisms by which both inflammation and thrombosis can lead to deleterious events after SAH, both of these pathological events have cross talk by which they potentiate each other (30). The overlap between inflammation and thrombosis, called thromboinflammation, has been reported to play roles in a number of brain diseases/damage, including ischemia (31–34). Since ischemia also plays a role in patient outcome as a downstream deleterious event from SAH, thromboinflammation may be an overlooked pathophysiology after SAH.

**Figure 1:**
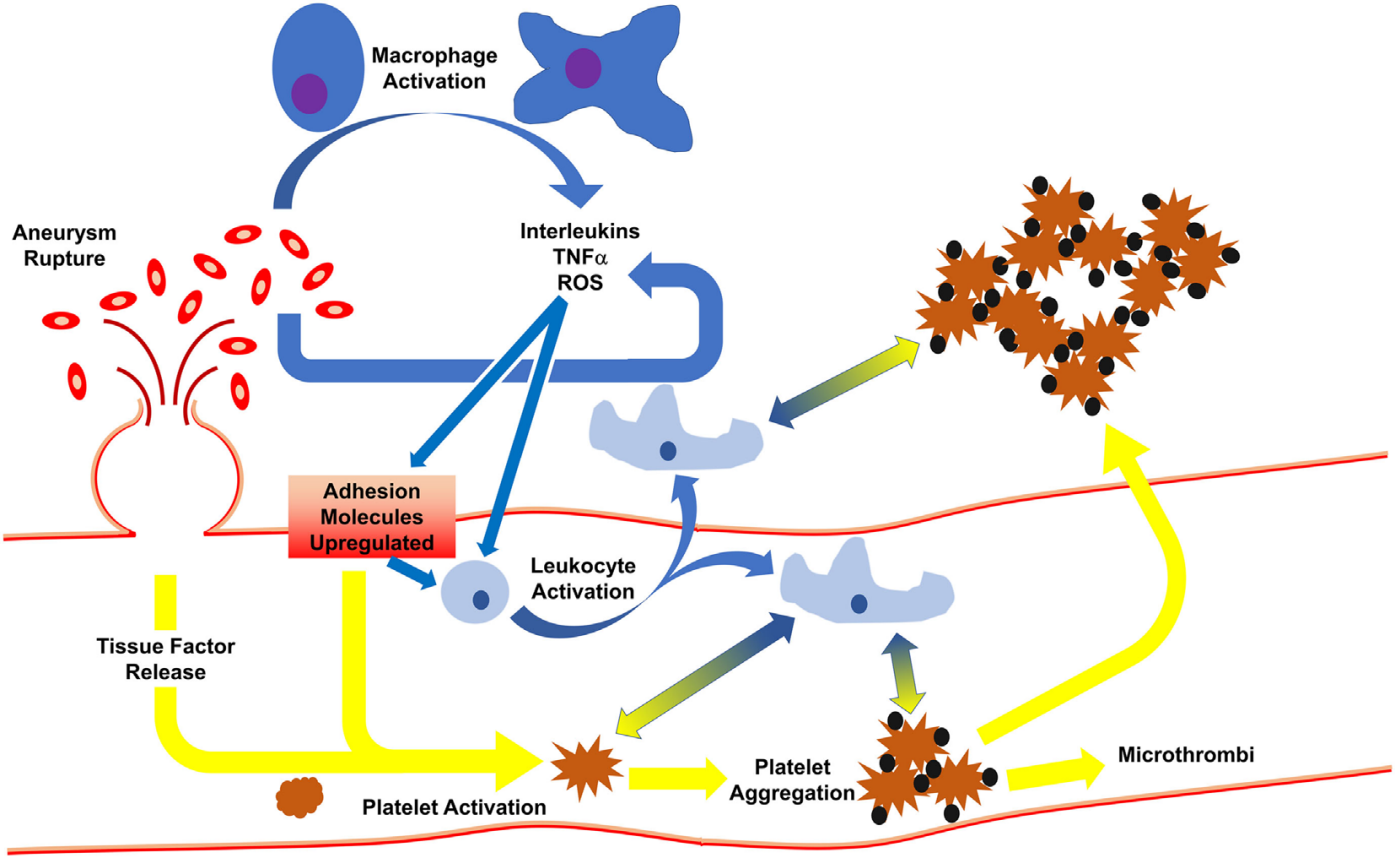
Initiation of inflammation and thrombosis after subarachnoid hemorrhage. Following rupture of an aneurysm, red blood cells spill into the subarachnoid space, activating macrophages which release cytokines. In response, endothelial cells express adhesion molecules to activate circulating leukocytes and platelets. This results in leukocyte infiltration, phagocytosis of red blood cells, and release of more cytokines. Activated platelets develop into thrombi which may travel downstream to occlude distal microvessels or may cross into the brain parenchyma. Platelet activation and aggregation signal for leukocyte activation, and vice versa, such that even when the aneurysm is treated, thromboinflammation continues *via* feedforward mechanisms.

Following SAH, activated macrophages release pro-inflammatory cytokines/chemokines for the recruitment of circulating macrophages to aid in RBC clearance (19). Phagocytosis of RBCs and subsequent death of the infiltrated leukocytes (occurring 2–4 days post-ictus) in the cerebrospinal fluid leads to release of a plethora of toxic molecules (35) including endothelins, oxygen free radicals, and hemoglobin/heme (and byproducts), thereby potentiating pro-inflammatory pathways and endothelial cell-mediated thrombosis. The latter event induces a positive feedforward loop, potentiating thromboinflammation by continuing to recruit systemic immune cells for debris clean-up, causing cell adhesion molecules to continuedly activate platelets, leading to microthrombi formation (36) (Figure 1).

Production/release of reactive oxygen species and nitric oxide adds to endothelial damage (37), stimulating endothelial expression of adhesion molecules (e.g., ICAM-1, P-selectin, VCAM, collagen, fibrinogen, etc.) (37, 38), and release of tissue factor and vWF, thereby activating platelets. This process is controlled by ADAMTS13 (a disintegrin-like metalloprotease with thrombospondin type 1 repeats-13), which acts to control clot formation by cleaving hyperactive ultralarge vWF to prevent platelet adherence (39). Interestingly, aSAH patients who go on to develop DCI are more hypercoagulable than aSAH patients who will not develop DCI (40, 41). Auxiliary to greater/sustained microthrombi formation, continued activation of coagulation by endothelial cells and platelets further promote inflammation *via* activation of circulating leukocytes through protease activated receptor-1 and toll-like receptor 4 signaling (36).

Finally, emerging evidence further displays the delicate balance that inflammation plays in modulating coagulation (36). In the non-diseased state, systemic leukocytes aid in preventing coagulation by expressing several anticoagulant factors (42–44). Yet, when pro-inflammatory signaling is initiated, leukocytes change phenotypes, thereby aiding in coagulation through the release of procoagulant factors (45, 46) and reduced expression and degradation of anti-coagulation factors (47–49). While leukocytes play a critical role in inducing and propagating inflammation, leukocytes also promote a hypercoagulable state after SAH (36) and may play a crucial role in DCI *via* thromboinflammation.

#### Clinical Evidence

To date, the exact role thromboinflammation plays in patients suffering from aSAH is not known. Yet, the following clinical studies suggest that thromboinflammation may be a key contributor to DCI after aSAH, and thus should be considered as a therapeutic target.

First, microthrombi have been reported as an associative cause of DCI and worse neurological outcome (50, 51). In human autopsy studies, microthrombi, reported to be throughout the brain, are associated with regions of infarct (50, 51), and also develop with a timing similar to DCI (6). Following aSAH, platelet activation is over-stimulated, resulting in microthrombi within small arterioles (51), peaking within 2 days and then again from 1 to 2 weeks post-ictus (50). Platelet-derived thromboxane B2 is higher and platelet count is lower in DCI patients compared to non-DCI patients (52, 53), suggesting platelet aggregation as a potential cause of microthrombi. In addition, aSAH patients are hypercoagulable with changes in platelet-activating factor (54), vWF (40, 54), and tissue plasminogen activator (55, 56) correlating with the incidence of DCI (54, 55) and poor patient outcome (28). Specific polymorphisms of plasminogen activator inhibitor (PAI) have higher PAI activity (57) leading to a higher incidence of DCI (41). Finally, in addition to an over active coagulation cascade, fibrinolysis after aSAH is impaired (40, 56). ADAMTS13, proposed as a critical link between inflammation and thrombosis (39), has been observed to have low activity after aSAH, and is associated with an increased risk of ischemic stroke (58) and may potentiate microthrombi formation. The inherent roles of leukocytes and platelets in inflammation and thrombosis, as well as the involvement of ADAMTS13, argue for thromboinflammation being considered as a therapeutic target after SAH.

Not only is the coagulation cascade not functioning properly, so too is inflammation continually hyperactive. Several pro-inflammatory cytokines have been linked with worse clinical outcome after aSAH (25–27, 59, 60). Increases in tumor necrosis factor α, interleukin-1α, interleukin-1β, interleukin-6, and interleukin-8 have all been found in the cerebrospinal fluid and blood (59, 61–68), with several factors also being associated with poor outcome and DCI (59, 61). Additionally, elevated adhesion molecules in the blood have also been observed after aSAH in patients, remaining elevated for 6–8 days, and are associated with DCI (69–71). Blood P-selectin is elevated in patients with DCI, suggesting that inflammation and platelet activation adhesion are associated with DCI (72, 73). Indeed, adhesion molecules are well documented to be at the crux of thromboinflammation (39, 74–76). These findings, which suggest thromboinflammation is active, are also supported by increased leukocyte infiltration into the brain following SAH (77–80) and also correlate with ischemia after SAH (81). Thus, the so-called “leukocyte–endothelial cell interaction” (78) may play a role in SAH pathophysiology (i.e., thromboinflammation).

A recent study on 106 aSAH patients showed that platelet activation and inflammation (*via* C-reactive protein measurement) occurred simultaneously and were associated with worse early brain injury and 3-month outcome (82). Similar to others (40, 54), Frontera et al. found that platelet activation and C-reactive protein levels were associated with DCI, but even more interesting, the authors reported that higher platelet activation and C-reactive protein levels at 72 h post-SAH correlate with DCI (82). The findings by Frontera et al. further support the potential role of thromboinflammation in DCI after aSAH, suggesting thromboinflammation may be an unexplored therapeutic target for improving aSAH outcome.

#### Evidence from Experimental Models

Thromboinflammation has also not been specifically studied in experimental models of SAH, nor has it been examined for correlation with microthrombi, ischemia, or DCI in animals. While DCI is unmeasurable in animals, several pathological measures are used as correlates for DCI (e.g., vasospasm, microthrombi formation, ischemia/infarct/cell death).

Several groups have reported the existence of microthrombi within the vasculature both locally, as well as distally from the SAH insult in experimental SAH models (83–86). In rodent models of SAH using autologous blood infusion, microthrombi and occluded blood vessels have been reported to occur throughout the brain at 2 days post-SAH (84) and can be observed for up to 7 days post-ictus (86). While microthrombi have been observed in puncture models of SAH (87, 88), it is the blood infusion models which offer the most insight into the potential role of thromboinflammation in clot formation after experimental SAH. Indeed, thrombi may be thrown downstream from the vessel rupture in endovascular perforation models as the mechanism by which microthrombi occur in the vasculature; however, no such vessel rupture is present in the autologous blood infusion models. In the blood injection SAH models, the likely cause of microthrombi formation and deposition within the cerebral vasculature distal from the SAH insult is inflammation.

Furthermore, following experimental SAH, endothelial damage resulting from leukocyte infiltration may lead to the downward spiral that is thromboinflammation. Indeed, post-SAH inflammation is devastating (19) and includes cytokine/chemokine upregulation (89, 90), increased expression of adhesion molecules (91, 92), and leukocyte infiltration (93–95). In fact, either depleting neutrophils or reducing neutrophil activity decreases microvascular injury following SAH in rats (93).

Similar to clinical findings, P-selectin has been observed to increase after experimental SAH, leading to activation of platelets and thrombi formation (84). Interestingly, platelet aggregates have been observed to extravasate into the brain parenchyma in the rodent SAH autologous blood injection model which likely propagates pro-inflammatory signaling (22), further showing that there is cross talk between inflammation and thrombosis after SAH.

Evidence from experimental models of SAH, similar to clinical findings, suggest that significant overlap between inflammation and thrombosis occurs post-SAH and may be involved in secondary injury and neurological deterioration. Future studies are warranted to investigate the exact role(s) thromboinflammation plays in SAH and its potential as a therapeutic target.

## Thromboinflammation as a Therapeutic Target to Prevent DCI

The detrimental roles thromboinflammation play in brain pathologies has led to thromboinflammation to be suggested as a therapeutic target (32, 96), but not for SAH. Based on the review presented above, thromboinflammation should also be considered a therapeutic target for patients of SAH (54). Potential targets of thromboinflammation may include expression of adhesion molecules, release of factors activating leukocytes and platelets simultaneously (e.g., selectins), disrupting platelet-leukocyte formations, and targeting ADAMTS13. To date, no clinical trials have studied these factors as specific therapeutic targets. Below we review some of the clinical trials which have targeted either coagulation or inflammation, and show that targeting these physiological events separately does not seem to hold the answer for SAH treatment.

### Previous Trials

#### Targeting Coagulation and Clot Clearance

Several early clinical trials investigated therapeutics directed at targeting coagulation following SAH, many of which used antiplatelet therapies. However, meta-analysis of antiplatelet therapy indicates that antiplatelet therapy has a trend for improving outcome in aSAH patients, with tendencies to decrease secondary ischemia (except for acetylsalicylic acid which shows no benefit) (97). Some of the antiplatelet therapies thus far investigated are acetylsalicylic acid, ticlopidine (adenosine diphosphate receptor inhibitor), dipyridamole (phosphodiesterase inhibitor), OKY-046/Calaclot (thromboxane synthetase inhibitor), a thromboxane A2 antagonist, and E5880 (inhibitor of platelet-activating factor receptor) (97). Of these agents, Calaclot and E5880 seemed to improve functional outcome, but additional studies need to be undertaken to determine the therapeutic benefit associated with antiplatelet therapies.

Studies have also examined the benefit of targeting clot clearance. In this regard, recombinant tissue plasminogen activator (administered intraventricularly) has been investigated in several clinical trials which have shown that although recombinant tissue plasminogen activator can improve clot clearance, it fails to attenuate cerebral vasospasm or DCI (98), reduce mortality (99), or improve functional outcome (100). These findings indicate that clot clearance alone may not be enough for aSAH.

#### Targeting Inflammation

Trials targeting inflammation have had varied success. Erythropoietin-β, despite having mechanisms of action against vasospasm and inflammation (101), had no effect on cerebral vasospasm, but did reduce DCI and improve functional outcome (102). This was a small study and is at high risk for bias (103), so additional trials are needed. Another anti-inflammatory agent, methylprednisolone, was found to improve functional outcome at 1 year despite having no effect on cerebral vasospasm or hypodensity on CT scans (104). It should be noted that methylprednisolone is an anti-inflammatory and thus may have no effect on vasospasm, although the lack of change in CT hypodensity is puzzling. Regardless, these trials suggest that inflammation indeed plays a major role in SAH outcome. Since there is considerable feedforward/back mechanisms between thrombosis and inflammation, anti-inflammatory agents may indirectly reduce thromboinflammation. While previous trials have shown promise, additional studies are needed to determine any benefit to patient outcome for anti-inflammatory agents as well as the relationship to thromboinflammation.

### Current Trials

The lack of promising data for therapeutics targeting either cerebral vasospasm (100, 103) or clot clearance suggest that SAH prognosis may lie within inflammation, platelet activation, or at the intersection of the two (i.e., thromboinflammation). To date, the only FDA approved treatment after aSAH is nimodipine. While the initial mechanism of action was purported to be *via* anti-vasospasm (105, 106), its effects seem to be more than just preventing vasospasm. Indeed, nimodipine can improve patient outcome irrespective of vasospasm attenuation (107). Although the exact mechanism of nimodipine is unknown, it may reduce microthrombosis, be neuroprotective, and inhibit cortical spreading ischemia (14, 108). Furthermore, nimodipine has been implicated as being anti-inflammatory (109), and it is important to note that calcium channel blockers may reduce leukocyte infiltration (110), and therefore reduce thromboinflammation. Mechanistic studies for the role(s) of nimodipine are needed to clarify the mechanisms by which nimodipine improves patient outcome after SAH. These studies may also shed light onto the therapeutic targets for thromboinflammation.

Fasudil is currently used in Asia instead of nimodipine, because the latter is not commercially available. Fasudil is a rho-kinase inhibitor which is reported to reduce hemodynamic dysfunction (i.e., vasoconstriction, endothelial injury) and inflammation through downstream signaling pathways *via* inhibition of rho-kinase (111). Initially, fasudil was used to target cerebral vasospasm, but fasudil was found to also reduce cerebral infarction, and improve functional outcome after aSAH (112). Thus, fasudil also seems to provide therapeutic benefit for aSAH patients, but still requires validation in large randomized controlled clinical trials (113). Being a rho-kinase inhibitor, fasudil may prevent a number of downstream signaling pathways involved in SAH pathophysiology [smooth muscle cell contraction and endothelial dysfunction (95, 114), inflammation, and leukocyte activation (115)], arguing that drugs with pleiotropic effects may be an answer. Similar to nimodipine, the exact mechanisms by which fasudil improves outcome following SAH remain a mystery, and translational studies for SAH will benefit for additional studies on the mechanism(s) of fasudil.

The recent study by Wessell et al. found that infusion of low-dose heparin administered with nimodipine is associated with increased odds of patient discharge (116), and, in a preliminary trial, heparin reduced cerebral vasospasm and infarct (117). These studies support the notion that combining therapies which reduce distinct injury mechanisms can provide increased effectiveness. Furthermore, these studies indicate that there may be therapeutic potential in anti-coagulation and anti-inflammation (103). Currently, heparin is currently being explored in the ASTROH trial (NCT02501434) which will examine the efficacy of heparin for reducing vasospasm and delayed neurological deficits in SAH patients.

Specific information regarding the role of thromboinflammation and its potential as a therapeutic target may be elucidated through the SoSTART trial (NCT03153150) and the etanercept (TNFα receptor antagonist) trial (NCT01865630). The SoSTART trial is set to investigate seven anti-coagulants for therapeutic benefit in patients of cerebral hemorrhage, including aSAH. These proposed drugs are known to be reduce coagulability and prevent inflammation (118, 119), which may reduce the overall thromboinflammation.

### Challenges in Targeting Thromboinflammation

Several challenges exist when considering thromboinflammation as a therapeutic target for preventing DCI. First and foremost, special care needs to be taken when identifying potential therapies for thromboinflammation since the ideal candidate will only target newly formed microthrombi and not have any significant effect on the clot formed at the site of aneurysm rupture. Once the aneurysm has been secured with clip or coil, the aneurysm is of less concern, but potential complications such as post-operative bleeding or bleeding along the ventriculostomy remain a risk.

In most pathologies, including SAH, both pro- and anti-inflammatory pathways are activated to promote debris/toxin clearance and repair of injured tissue, respectively. Since pro-inflammatory cytokines and downstream signaling is linked with thromboinflammation (34), it is critical to pursue agents which mitigate pro-inflammatory cytokines. So far research has largely focused on this, however, anti-inflammatory signaling aids in repair and healing, thus this arm of inflammation should remain active (or upregulated) after reducing thromboinflammation.

Finally, although experimental studies are required to understand pathophysiology, uncover signaling mechanisms, and investigate novel drugs and potential therapeutic targets, animals (including non-human primates) have differences when it comes to coagulation (120, 121), as well as inflammation (122, 123). Therefore, it follows that animals may have distinct differences when it comes to thromboinflammation and its treatment. Future studies should examine these differences to aid in therapeutic development against thromboinflammation.

## Conclusion

Thromboinflammation, the cross talk between the thrombotic and inflammatory pathways, likely plays a critical role in the development of DCI after SAH, thereby having significant impact on overall patient outcome. The current understanding of thromboinflammation and its role in SAH pathophysiology is only beginning to emerge, but evidence from experimental and clinical studies suggest that preventing thromboinflammation has the potential for benefiting patients of aSAH.

## Author Contributions

All authors contributed to the conception and drafting of this article.

## Conflict of Interest Statement

The authors declare that the research was conducted in the absence of any commercial or financial relationships that could be construed as a potential conflict of interest.
